# Exploring shared therapeutic targets for Alzheimer’s disease and glioblastoma using network pharmacology and protein-protein interaction approach

**DOI:** 10.3389/fchem.2025.1549186

**Published:** 2025-03-12

**Authors:** Sushma Pradeep, M. R. Sai Chakith, S. R. Sindhushree, Pruthvish Reddy, Esther Sushmitha, Madhusudan N. Purohit, Divya Suresh, Nanjunda Swamy Shivananju, Ekaterina Silina, Natalia Manturova, Victor Stupin, Shiva Prasad Kollur, Chandan Shivamallu, Raghu Ram Achar

**Affiliations:** ^1^ Department of Biotechnology and Bioinformatics, School of Life Sciences, JSS Academy of Higher Education and Research, Mysuru, Karnataka, India; ^2^ Centre for Digital Health and AI, JSS Medical College, JSS Academy of Higher Education and Research, Mysuru, Karnataka, India; ^3^ Department of Pharmacology, JSS Medical College, JSS Academy of Higher Education and Research, Mysuru, Karnataka, India; ^4^ Department of Biotechnology, Acharya Institute of Technology, Bengaluru, Karnataka, India; ^5^ Department of Pharmaceutical Chemistry, JSS College of Pharmacy, JSS Academy of Higher Education and Research, Mysuru, Karnataka, India; ^6^ Department of Biotechnology, JSS Science and Technology University, Sri Jayachamarajendra College of Engineering, Mysuru, Karnataka, India; ^7^ Institute of Digital Biodesign and Modeling of Living Systems, I. M. Sechenov First Moscow State Medical University (Sechenov University), Moscow, Russia; ^8^ Department of Surgery, Pirogov Russian National Research Medical University, Moscow, Russia; ^9^ School of Physical Sciences, Amrita Vishwa Vidyapeetham, Mysuru Campus, Mysuru, Karnataka, India; ^10^ Division of Biochemistry, School of Life Sciences, JSS Academy of Higher Education and Research, Mysuru, Karnataka, India

**Keywords:** Alzheimer’s disease, glioblastoma, brain cancer, network pharmacology, protein-protein interaction, *Eclipta alba*, molecular docking, and molecular docking simulation

## Abstract

**Background:**

Alzheimer’s disease (AD) and glioblastoma (GBM) are complex neurological disorders with distinct pathologies but overlapping molecular mechanisms, including neuroinflammation, oxidative stress, and dysregulated signaling pathways. Despite significant advancements in research, effective therapies targeting both conditions remain elusive. Identifying shared molecular targets and potential therapeutic agents could offer novel treatment strategies for these disorders.

**Methodology:**

The study employs an integrative network pharmacology approach to explore the therapeutic potential of bioactive compounds from *Eclipta alba*, a medicinal herb known for its neuroprotective and anti-inflammatory properties. A systematic methodology was adopted, starting with network pharmacology analysis using STRING and DisGeNET databases, which identified 617 common genes associated with AD and GBM. Among these, key hub genes—TP53, STAT3, AKT1, and IL6—were prioritized using Cytoscape for network visualization and analysis.

**Results:**

Molecular docking studies were conducted using PyRx software to assess the binding interactions of 26 phytochemicals from Eclipta alba against the identified target genes. Luteolin exhibited the highest binding affinity to IL6 (−7.8 kcal/mol), forming stable hydrogen bonds and hydrophobic interactions. To further validate this interaction, molecular dynamics simulations (MDS) were performed using GROMACS, confirming the stability of the Luteolin-IL6 complex. Additionally, MM-PBSA binding energy calculations using AmberTools (−145.44 kJ/mol) provided further evidence of a strong and stable interaction. Pharmacokinetic and toxicity evaluations, conducted using SwissADME and pkCSM, highlighted luteolin’s favorable drug-like properties, including good bioavailability and low toxicity. These findings suggest that luteolin may serve as a promising multi-target therapeutic agent for AD and GBM by modulating key pathological pathways.

**Conclusion:**

The present study provides a strong computational foundation for further *in vitro* and *in vivo* validation. The results highlight the potential of luteolin in developing dual-target treatment strategies for neurodegenerative and oncological disorders, offering new avenues for therapeutic advancements.

## 1 Introduction

**FIGURE 1:**
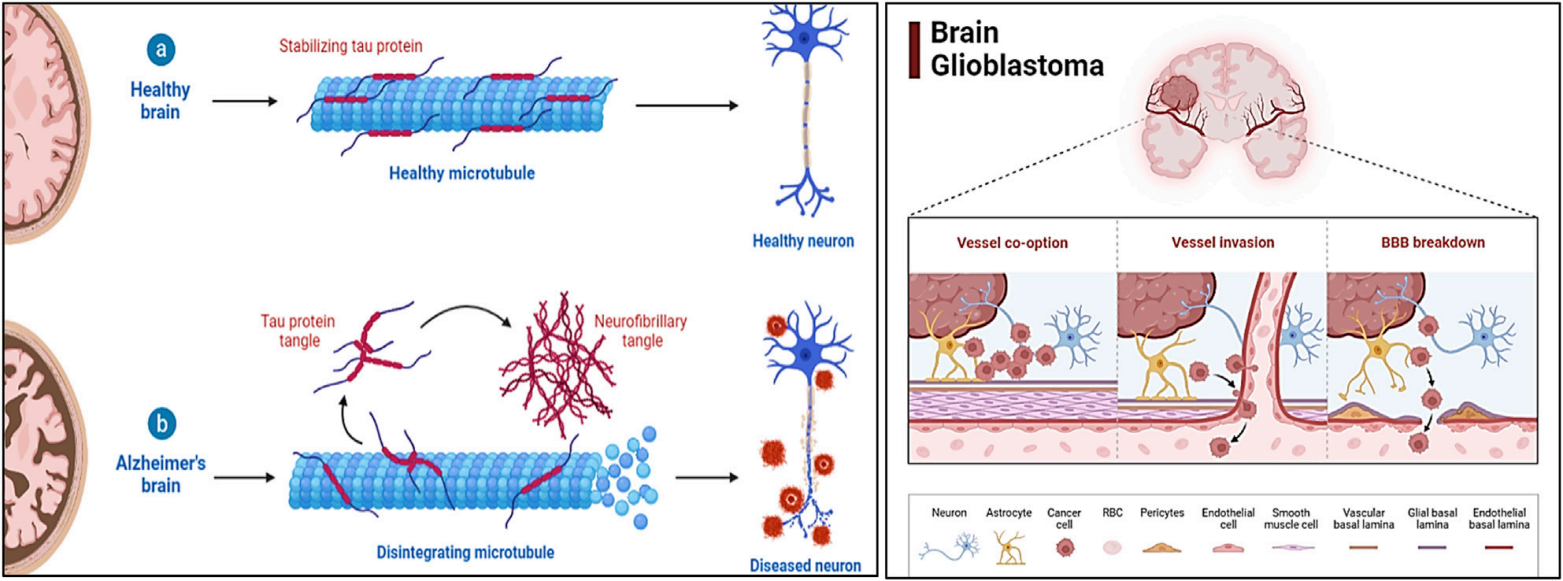
The schematic representation illustrates the multifactorial pathology of AD and GBM, highlighting key molecular and cellular mechanisms. For AD, it depicts amyloid-beta plaque accumulation, tau hyperphosphorylation, neuroinflammation, oxidative stress, and mitochondrial dysfunction, leading to neurodegeneration. For GBM, it showcases uncontrolled proliferation, angiogenesis, immune evasion, and resistance mechanisms driven by EGFR overactivation, PI3K/Akt/mTOR signaling, and tumor microenvironment interactions.

Alzheimer’s Disease (AD) and glioblastoma (GBM) represent two of the most challenging health conditions, each associated with significant morbidity and mortality. AD is a progressive neurodegenerative disorder that primarily affects cognitive and memory functions, resulting from the accumulation of abnormal amyloid-beta plaques and tau neurofibrillary tangles in the brain (Pradeep S et al., 2020). It is the most common cause of dementia globally, with cases projected to rise dramatically in the coming decades. GBM, on the other hand, is the most aggressive and lethal form of brain cancer, characterized by rapid growth, high invasiveness, and poor prognosis despite advanced therapeutic interventions (Figure 1). While these diseases appear distinct in etiology and pathology, recent studies have highlighted overlapping molecular and pathological mechanisms, offering new insights into their potential interconnection. AD and GBM both involve intricate interactions of genetic, molecular, and cellular factors. Common features such as chronic inflammation, oxidative stress, aberrant signaling pathways, and disruptions in the cellular microenvironment have been implicated in both conditions. Key molecular players, including tumor protein p53 (TP53), signal transducer and activator of transcription 3 (STAT3), AKT serine/threonine kinase 1 (AKT1), and interleukin-6 (IL6), are known to contribute to neurodegeneration in AD and tumorigenesis in GBM. Understanding the shared pathways and identifying therapeutic targets that modulate these processes could lead to innovative strategies to treat or manage both diseases (Burhan et al., 2016).

Computational methodologies have emerged as powerful tools in of discovering and developing therapeutic agents. Network pharmacology, an approach that integrates systems biology with drug design, provides a framework to analyze the complex interactions between genes, proteins, and small molecules (Lenderking et al., 2014) Protein-protein interaction (PPI) network analysis further enables the identification of critical hubs or nodes that play central roles in disease progression. By applying these methods, researchers can prioritize key targets for experimental validation and drug development. The convergence of AD and GBM pathologies provides a unique opportunity to explore multi-target therapeutic strategies. Traditional single-target drug development approaches often fail to address the multifactorial nature of these diseases. Thus, the study aims to identify common genetic and molecular targets that can be modulated to simultaneously mitigate the effects of AD and GBM. Natural products, particularly phytocompounds from medicinal plants like *Eclipta alba*, have shown promise in addressing complex diseases due to their multi-target properties, low toxicity, and bioavailability (Pradeep et al., 2021).

*Eclipta alba*, commonly known as false daisy or bhringraj, is a herb widely used in traditional medicine for its neuroprotective, anti-inflammatory, and antioxidant properties. This plant is rich in phytochemicals, including flavonoids, alkaloids, terpenoids, coumestans, and polyphenols, which have demonstrated significant pharmacological effects in various preclinical studies. It has been traditionally used in Ayurvedic and Chinese medicine to treat a wide range of conditions, including liver disorders, hair loss, skin diseases, and immune-related conditions. *Eclipta alba* is also known for its hepatoprotective, antimicrobial, antiulcer, and anti-aging effects, making it a versatile medicinal plant (Pradeep et al., 2020). The plant’s bioactive compounds also possess properties that facilitate blood-brain barrier (BBB) permeability, a critical factor for treating central nervous system (CNS) disorders like AD and GBM. Preclinical studies have demonstrated that *E. alba* extracts can enhance cognitive function, reduce neurodegeneration, and exhibit cytotoxic effects on cancer cells, further supporting its utility in addressing these diseases (Uppar et al., 2021). Ubiquitous in plants, flavonoids and related polyphenolic compounds have garnered significant attention for their diverse biological activities, including antioxidant, anti-inflammatory, and anti-cancer properties. These effects stem from their ability to modulate various cellular signaling pathways and interact with multiple molecular targets. This study leverages network pharmacology to identify shared therapeutic targets between AD and GBM, two conditions sharing overlapping pathological mechanisms such as neuroinflammation, oxidative stress, and dysregulated signaling, including disruptions in amyloid-beta processing (Ramanan et al., 2016). Luteolin, a naturally occurring flavonoid within this class, and its isomers, including apigenin, chrysin, diosmetin, and luteolinidin, are of particular interest due to their diverse biological activities and potential for therapeutic intervention. By identifying these common vulnerabilities, we aim to uncover potential therapeutic strategies that could be effective in treating both devastating diseases, potentially leading to the development of novel multi-target drugs (Sudarshan et al., 2019).

The study employed an integrated computational approach to identify and evaluate therapeutic targets and candidate compounds. Databases such as DisGeNET and STRING were used to identify genes common to AD and GBM.PPI network analysis was conducted using Cytoscape, a platform for visualizing molecular interaction networks. Key targets were prioritized based on topological parameters such as degree centrality, closeness centrality, and betweenness centrality. To further validate the identified targets, molecular docking (MD), ADMET (absorption, distribution, metabolism, excretion, and toxicity) analysis, molecular dynamic simulations (MDS) andbinding energy calculations, studies were conducted (Pradeep et al., 2022). These computational techniques provided insights into the binding affinity, stability, and interaction profiles of selected phytocompounds with the target proteins.

Unlike previous research that focused on either AD or GBM separately, this study explores their shared pathological mechanisms—inflammation, oxidative stress, and dysregulated signaling pathways—to identify common molecular targets that can be modulated for a multi-target treatment strategy through network pharmacological studies. the present study bridges traditional medicine with modern drug discovery by scientifically validating the neuroprotective and anticancer properties of *E. alba* using computational biology. It represents a paradigm shift in drug discovery by demonstrating how network pharmacology, molecular docking, molecular dynamics, and machine learning-based ADMET predictions can efficiently prioritize multi-target natural compounds for treating complex diseases. By uncovering the dual therapeutic potential of luteolin, this study provides a novel framework for repurposing medicinal plants in neurodegeneration and oncology, paving the way for future experimental validation and clinical translation. The integration of systems biology with advanced computational techniques represents a paradigm shift in drug discovery, particularly for diseases like AD and GBM that have historically been challenging to treat. Ultimately, the study aims to elucidate the therapeutic potential of *E. alba* phytochemicals in addressing the shared molecular mechanisms of AD and GBM.

## 2 Materials and methods

### 2.1 Prediction of targeted genes and selection of common genes

**FIGURE 2:**
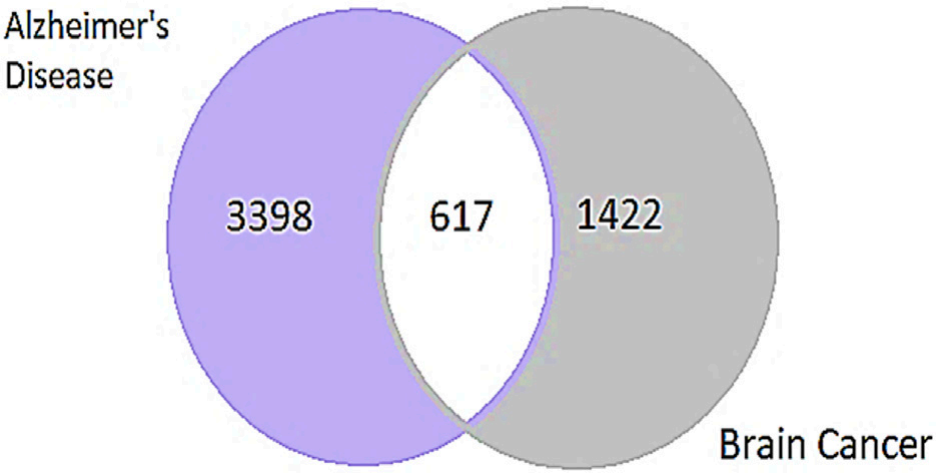
Representing the number of common genes from AD and Brain Cancer through a Venn diagram. The two circles represent the distinct gene sets associated with AD and brain cancer, with the overlapping region indicating the number of common genes involved in both conditions.

The study used the DisGeNET database (https://www.disgenet.org/search) to identify genes associated with AD and GBM. The search used the keywords “AD” and “Brain Cancer” and applied the filter for “*Homo sapiens*” (human). The results showed a total of 3,398 genes linked to AD and 1,422 genes associated with brain cancer. After identifying genes associated with AD and GBM using the DisGeNET database, the relevant genes were extracted from Microsoft Excel. Subsequently, the focus was narrowed down to 617 genes that were commonly involved in both diseases (Prasad et al., 2020c) (Figure 2).

### 2.2 Construction of PPI and C-T network

The STRING web tool (https://string-db.org) was utilized to construct a protein-protein interaction network for the genes that were found to be common to both AD and GBM. The network obtained from the STRING database was imported into Cytoscape 3.8.2. This allowed for the identification of the most relevant gene among the 617 genes common to both AD and GBM. Firstly, ensuring that the STRING data had been successfully retrieved in Cytoscape 3.8.2 was necessary. Next, the network constructed using the STRING database was imported. Subsequently, individual networks were created based on the p-values of the genes using MCODE, a plugin installed in Cytoscape (Chen et al., 2012).

### 2.3 Computer aided drug discovery

The objective of protein-ligand interaction analysis is to identify the most favorable binding interactions between a small molecule (ligand) and a protein. RMSD values are calculated for all computationally generated poses of potential protein-ligand bindings (Khandagale et al., 2022).

#### 2.3.1 Protein preparation and validation

**FIGURE 3:**
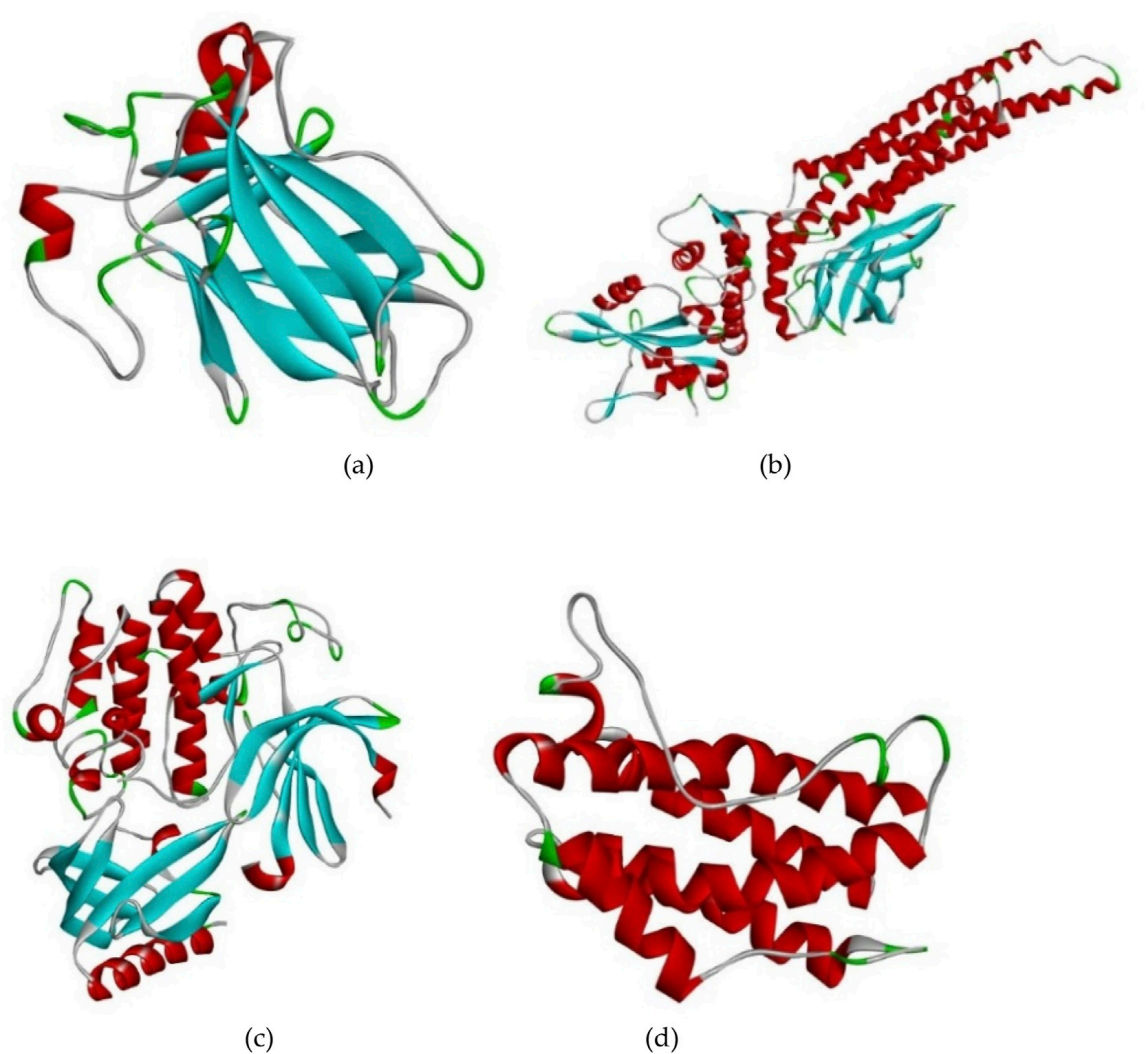
The three-dimensional representation of Four Protein structures selected for molecular interaction studies **(A)** 1TUP, **(B)** 6TLC, **(C)** 6S9X, and **(D)** 1IL6.

Among the top 15 genes, only four are being finalized. The proteins encoded by each gene are being selected, considering only those that play a role in AD and GBM pathology. The considered genes are TP53, STAT3, AKT1, and IL6. Following the network analysis, the most significant gene, TP53 was selected, for docking investigation, followed by Signal Transducer and Activator of STAT3, AKT1, and IL6. The structural features of the chosen protein molecules were obtained from RCSB PDB (Protein Data Bank) (https://www.rcsb.org/) (Majd et al., 2019) (Figure 3).

The proteins 1IL6, 1TUP (Tumor suppressor P53 complexed with DNA), 6TLC (Unphosphorylated human STAT3), 6S9X, and 1IL6 are primarily involved in AD and brain tumors. Neuro-inflammation is another biological process associated with AD etiology, often characterized by activated microglia cells and increased expression of cell-surface proteins and cytokines like interleukin-6 in response to Aβ deposition (IL-6). Systemic inflammation has been linked to neurodegeneration, with elevated interleukin-6 levels associated with an increased risk of dementia. Endothelial cells of the blood-brain barrier (BBB) are expected to play a role in neuropathology associated with systemic inflammation. Protein toxicity is a fundamental feature of most types of dementia, including AD with Lewy bodies and frontotemporal dementia (Prasad et al., 2020b). These clumps are formed when proteins misfold and aggregate, causing harm to brain cells. The protein 1TUP can contribute to various malignancies, including brain tumors, as it encodes TP53 genes, frequently altered in human astrocytoma and glioblastoma samples. The protein 6TLC is encoded by the STAT3 gene, which belongs to the STAT protein family and has been linked to the regulation of synaptic plasticity, cognition, and cognitive deficiencies caused by hTau accumulation (Lenderking et al., 2014).

The 3-dimensional structures of each protein were retrieved from the RCSB PDB database (https://www.rcsb.org) using their protein IDs. PDB is a database that contains three-dimensional structural data for large molecules such as proteins and nucleic acids (Kollur et al., 2021). The proteins downloaded from the PDB database needed to be cleaned for the docking method. Specifically, the proteins 1TUP, 6TLC, 6S9X, 1IL6, and 6ZFL were downloaded in the PDB format. If a protein contained repeated chains, the extra chains were deleted. Additionally, water molecules present in the protein structures were removed. To confirm the quality of the protein structure, the protein file was uploaded to PROCHECK RAMPAGE, and the program was run (Sharma et al., 2021).

#### 2.3.2 Protein active site prediction

**FIGURE 4:**
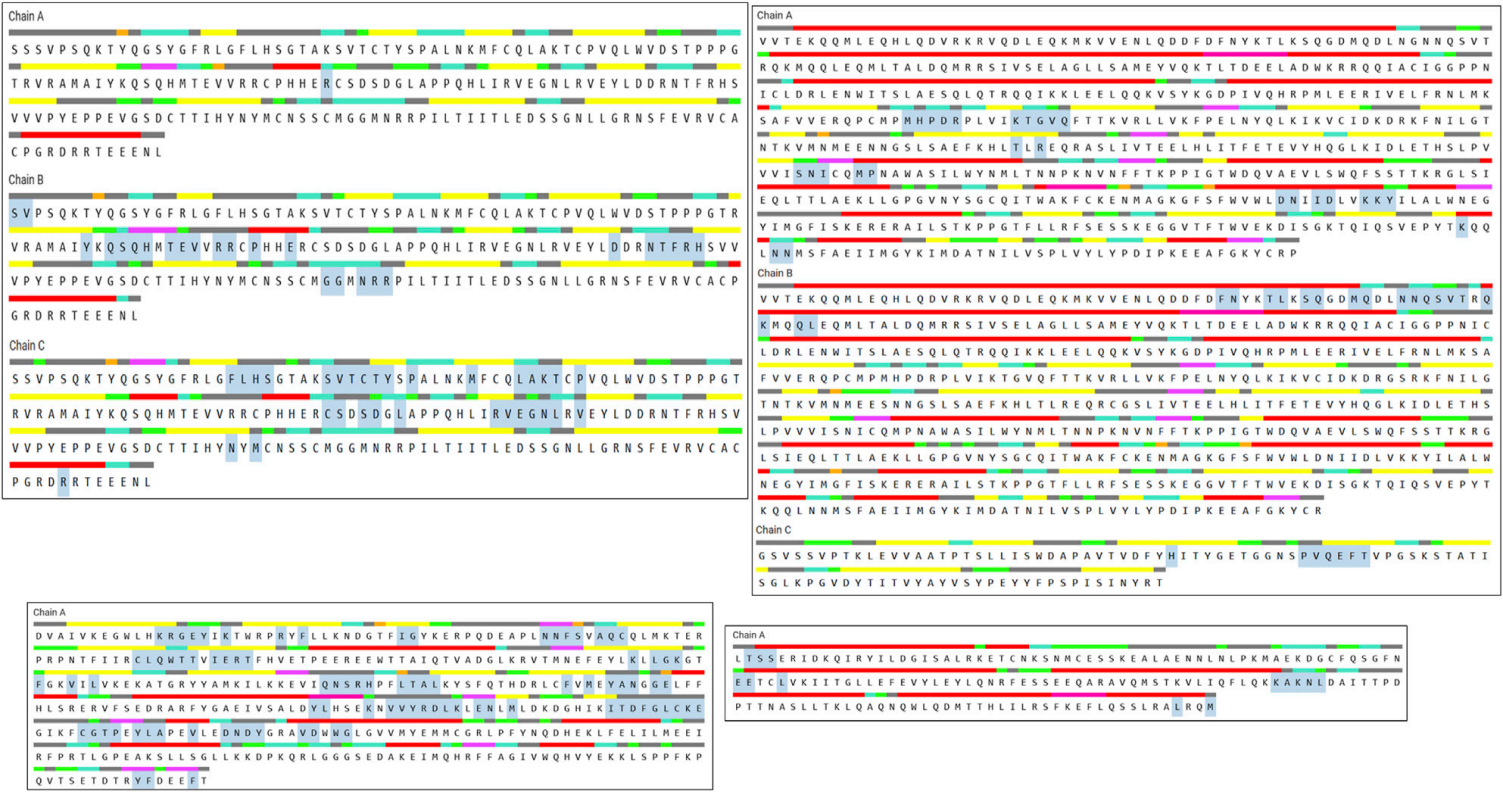
The binding site residues details of four selected proteins and the respective amino acid residues are highlighted in grey. These residues play a crucial role in facilitating ligand-protein interactions, contributing to the overall binding stability and specificity.

The subsequent step involves selecting the active site or active amino acids within the protein. The active site of a specific protein can be determined using CASTp (Computed Atlas of Surface Topography of Proteins) (http://sts.bioe.uic.edu). The cleaned protein can be uploaded as a file to CASTp, and results can be obtained after a few minutes. The specific active sites within the amino acids of the given protein can be readily identified (Sajal et al., 2022) (Figure 4).

#### 2.3.3 Construction of ligand library preparation and optimization

The phytocompounds of *E. alba*, were retrieved from the IMPPAT (Indian Medicinal Plants, Phytochemistry, and Therapeutics) database (https://cb.imsc.res.in/imppat/basicsearchauth). This comprehensive database contains information on over 1700 Indian medicinal plants, each associated with 1100 different therapeutic applications. The Phytocompound which passed Lipinski’s rule of 5 was considered and its.pdb files were downloaded from the IMPPAT database and they are represented in the below Table 1 (Pushpa et al., 2022).

**TABLE 1:** ligand table which has screened in all the parameters of *Eclipta alba* which is taken by using IMPPAT database.

Sl. No	Phytocompound name	Molecular weight (g/mol)	Lipinski’s rule of 5	Structure
1	4beta-Hydroxyverazine	413.65	Passed	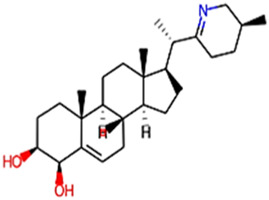
2	25beta-Hydroxyverazine	413.65	Passed	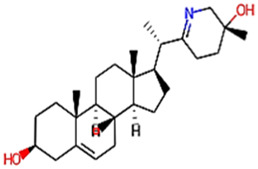
3	Veramiline	399.66	passed	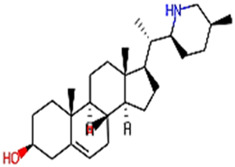
4	Linoleic acid	280.45	passed	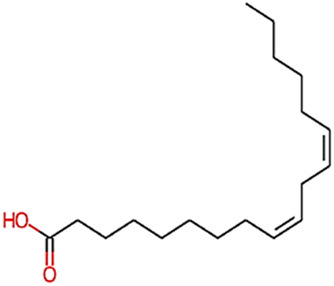
5	Palmitic acid	256.43	passed	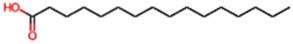
6	Ricinoleic acid	298.47	passed	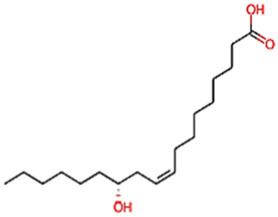
7	Nicotine	162.24	passed	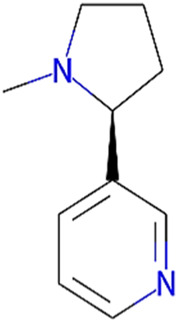
8	Wedellactone	314.25	Passed	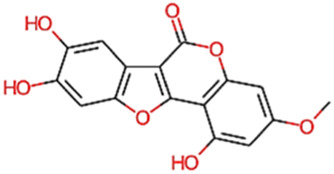
9	beta-Amyrin	426.73	Passed	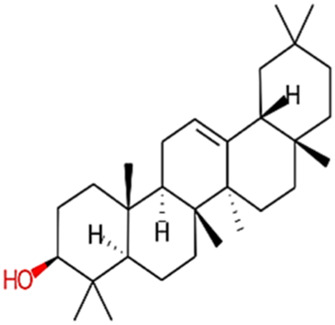
10	beta-Sitosterol	414.72	Passed	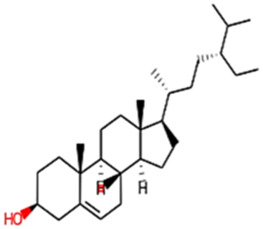
11	2-Formyl-terthienyl	278.42	Passed	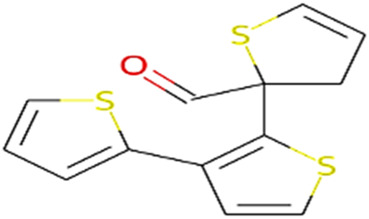
12	alpha-Terthienylmethanol	278.42	Passed	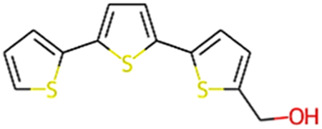
13	Oleic acid	282.47	Passed	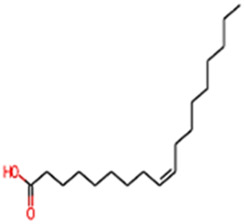
14	Demethylwedelolactone	300.22	Passed	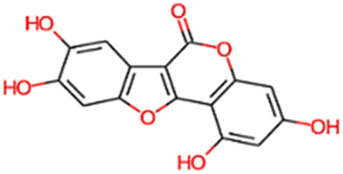
15	Ecliptalbine	409.61	Passed	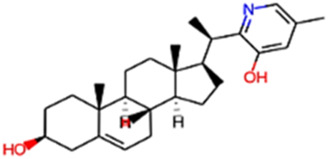
16	Pratensein	300.27	Passed	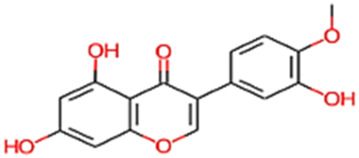
17	Stigmasterol	412.7	Passed	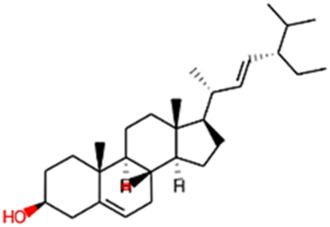
18	beta-Sitosterol	414.72	Passed	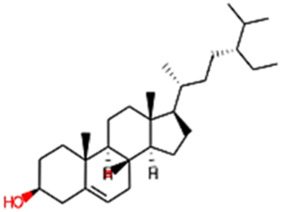
19	beta-Amyrin	426.73	Passed	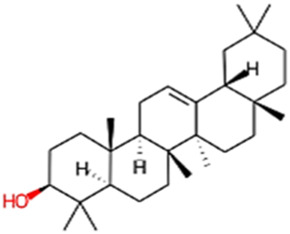
20	Ursolic acid	456.71	Passed	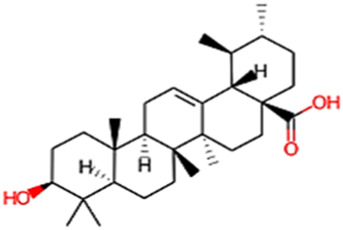
21	Oleanolic acid	456.71	Passed	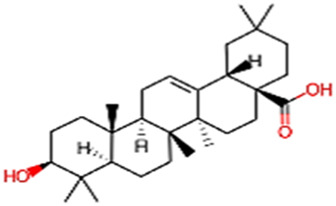
22	beta-Farnesene	204.36	Passed	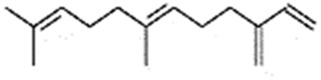
23	Pratensein	300.27	Passed	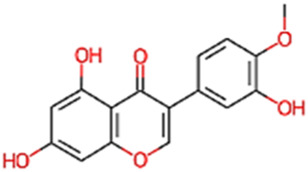
24	Diosmetin	300.27	Passed	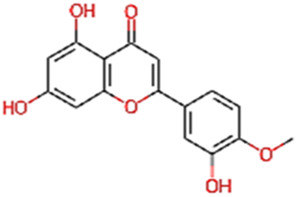
25	4-Hydroxybenzoic acid	138.12	Passed	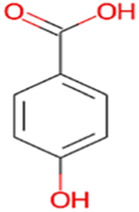
26	Luteoline	286.24	Passed	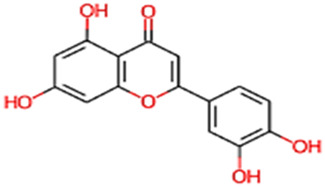

#### 2.3.4 Molecular docking simulation

PyRx (https://pyrx.sourceforge.io) is a computational drug discovery software that enables the screening of chemical libraries against potential therapeutic targets. To initiate the molecular docking process with PyRx, the first step is to load the protein, subsequently, all 26 ligands that have been cleaned and prepared using the ArgusLab tool should be imported using the “clean geometry” option (Jain et al., 2021). This is the procedure for performing molecular docking with PyRx, as previously described. Initially, one by one the cleaned protein is imported, followed by the importation of all 26 ligands from the *E. alba* plant. Later in the process, the protein will be transformed into a macromolecule, and the phytochemical substances will be converted into ligands. All the active sites within the imported protein need to be selected in PyRx. Then, the AutoDock Vina wizard should be set up and initiated again, with all 26 ligands and their respective molecules selected. The process proceeds with the “forward” option, and results are obtained in the form of a table. Each ligand will have a total of 8 conformations with the respective protein molecule. Consequently, the results will include binding affinity (kcal/mol), mode, RMSD lower bound, and RMSD upper bound for all 26 ligands with respect to the protein. The selection of ligands should be based on their binding affinity (Aisen et al., 2022). Typically, binding affinity results are expressed as negative values. A least binding affinity indicates strong and favorable binding of that ligand with the respective protein molecule. Additionally, among the 8 conformations, the top 2 confirmations can be considered further as they exhibit the least binding energy and RMSD (Pradeep et al., 2022).

The results of the molecular docking protocol indicated its suitability for screening phytocompounds. Active compounds were subsequently selected for further investigation based on the C-T network. The virtual screening results for a total of 26 ligands (compounds) evaluated for investigation are presented in the table below, which displays their binding affinity (kcal/mol), the number of hydrogen bonds, and non-binding interactions with the protein molecule (Behl et al., 2022). These compounds were visualized and analyzed using Discovery Studio, a software package for simulating small molecule and macromolecule systems.

### 2.4 Pharmacokinetic properties

The ADMET analysis of Luteolin and Wedelolactone was conducted using the pkCSM tool (https://biosig.lab.uq.edu.au/pkcsm/) a computational platform designed for predicting pharmacokinetic and toxicity properties of compounds based on their molecular structure. The study assessed absorption, distribution, metabolism, excretion, and toxicity profiles to understand the drug-likeness and safety of these compounds (Berte et al., 2018).

### 2.5 Molecular dynamic simulation

Molecular dynamics simulations were performed using the GROMACS simulation software. The simulations involved running MD simulations for the complexes with the highest affinities in water using the CHARMM36 force field (Ankegowda et al., 2020). CHARMM36 typically uses an all-atom model, meaning that each atom in the molecule (including hydrogens) is explicitly represented in the simulation. This level of detail provides a more accurate description of the interactions within the system. These simulations were carried out for a duration of 50 ns, with trajectory and energy files recorded every 10 ps. To solvate the system, a truncated octahedral box containing TIP3P water molecules was employed. The protein was centered in the simulation box, maintaining a minimum distance of 1 nm from the box edge to satisfy the minimum image convention. To neutralize the entire system, 4 potassium ions were added to the protein complexes (Bogdan et al., 2020; Reddy et al., 2023). To prevent steric collisions, a minimization step was performed using the steepest descent method, running for 5000 steps until convergence was achieved within a maximum force of 1000 (KJ mol^−1^ nm^−1^). To ensure a properly converged system for the production run, all three systems underwent equilibration at NVT (constant number of particles, volume, and temperature) and NPT (constant number of particles, pressure, and temperature) ensembles for 100 ps (50,000 steps) and 1000 ps (1,000,000 steps), respectively, utilizing time steps of 0.2 and 0.1 fs at 300 K. The production run of the simulation was conducted at a constant temperature of 300 K and pressure of 1atm (NPT) using the Parrinello-Rahman and weak coupling velocity-rescaling methods (Cheng et al., 2010). Before analyzing the results of MD simulation, it's essential to ensure that the system has reached equilibrium, which means system’s properties (temperature, pressure, energy) are stable and no longer changing significantly over time. In this study, 50 ns provided sufficient time for the system to equilibrate, especially after the initial minimization and equilibration steps.

### 2.6 Binding energy calculations

With the help of the Molecular Mechanics/Poisson-Boltzmann Surface Area (MM-PBSA) method, binding free energy calculations were performed on the results of the MDS run for luteolin of the target protein 1IL6. To ascertain the degree of ligand interaction with protein, molecular dynamics simulations and thermodynamics are once again used (Prasad et al., 2021). The binding free energy for each ligand-protein combination was calculated using the gmmpbsa software and MmPbStat.py script, which takes the GROMACS 2018.1 trajectories as input (Prasad et al., 2021). The binding free energy is determined by the g mmpbsa program using three different factors: molecular mechanical energy, polar and a polar solvation energies, and molecular mechanical energy. Using MDS, the calculation is completed. To compute ∆G with dt 1000 frames, the latest 100 ns of trajectory were taken into consideration. It is assessed using polar and a polar solvation energies, as well as molecular mechanical energy (Chandrashekar et al., 2022). Below are the Formulas 1, 2 that are used to determine the free binding energy.

$${∆G}_{Binding}=G_{Complex}-\left(G_{Protein}+G_{Ligand}\right)$$ (1)

$$∆G={∆E}_{MM}+∆G_{\text{Solvation}}-T∆S=∆E_{\left(Bonded+nonbonded\right)}+{∆G}_{\left(Polar+nonpolar\right)}-T∆S$$ (2)

GBinding: binding free energy, GComplex: total free energy of the protein-ligand complex, GProtein and GLigand: total free energies of the isolated protein and ligand in solvent, respectively, ∆G: standard free energy, ∆EMM: average molecular mechanics potential energy in vacuum, ∆GSolvation: solvation energy, ∆E: total energy of bonded as well as non-bonded interactions, ∆S: change in entropy of the system upon ligand binding, T: temperature in Kelvin.

## 3 Results

### 3.1 Construction and analysis of PPI and C-T network

**FIGURE 5:**
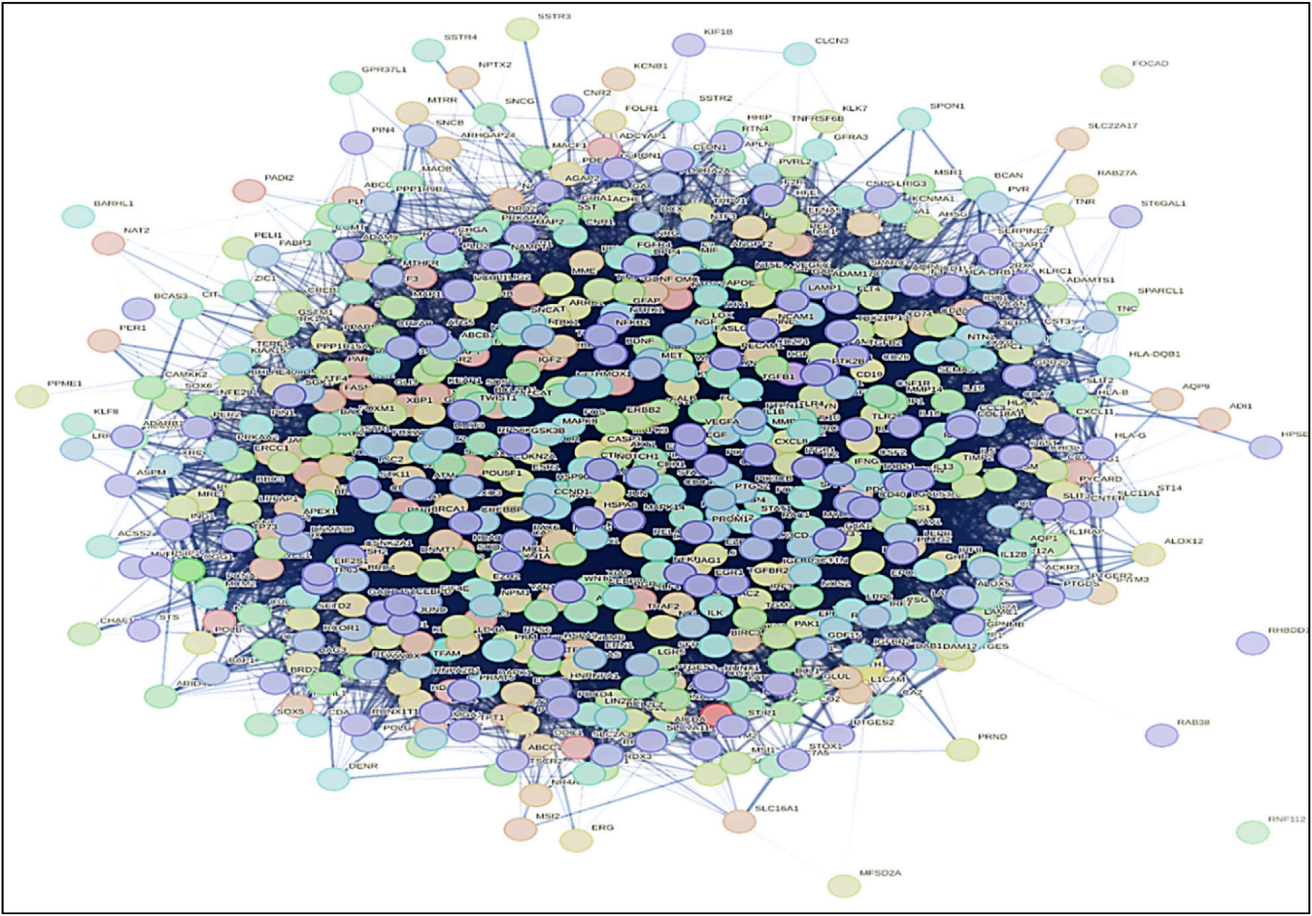
PPI network of common genes of both AD and GBM was built by using STRING database. The network visualization reveals key hub genes and interaction clusters, highlighting potential functional links between neurodegeneration and tumorigenesis. Identifying highly connected nodes in the PPI network helps in prioritizing therapeutic targets for drug repurposing and combination therapies.

The identification and refinement of common genes between AD and GBM followed a systematic computational approach. Initially, gene datasets were retrieved from the DisGeNET database, this resulted in a total of 3,398 genes associated with AD and 1,422 genes linked to GBM. To determine the common genes between these two diseases, a comparative analysis was performed using Microsoft Excel to filter shared genes and Venn diagram-based intersection analysis leading to the identification of 617 common genes (Figure 5).

Once the 617 overlapping genes were identified, further screening was carried out to remove redundant or non-relevant genes through a network pharmacology approach. The list of common genes was input into the tool, and the network was generated. Interactions were established using a confidence level of 0.09, and the organism filter was set to *H. sapiens*, ensuring that dissociation targets were excluded from the target genes. In summary, the interaction network was constructed using the STRING database.

**FIGURE 6:**
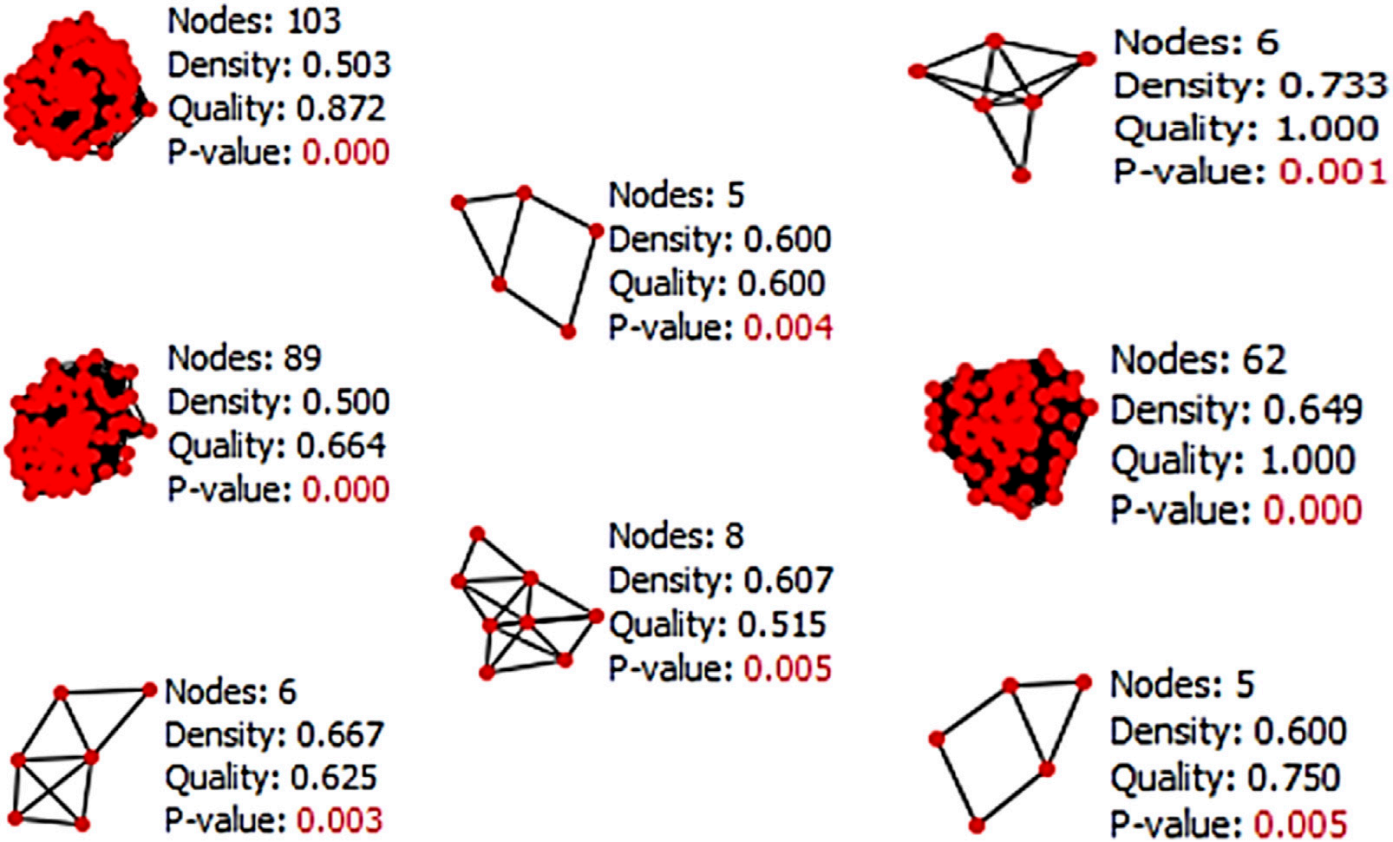
Result of Cytocluster representing the clusters which contain p-value less than 0.05 from the whole network.

The Cytocluster plugin offers various algorithms that can be utilized to determine the p-value of each cluster. The analysis employed the CLUSTER-ONE algorithm, which involves clustering with overlapping neighborhoods. CLUSTER-ONE is a graph clustering algorithm specifically designed to handle weighted graphs and generate overlapping clusters (Shivalingaiah et al., 2022a). In total, 13 clusters were obtained from the entire network based on their p-values, but only 8 clusters with a p-value less than 0.05 were considered for subsequent analysis (Figure 6).

### 3.2 Gene prediction

**FIGURE 7:**
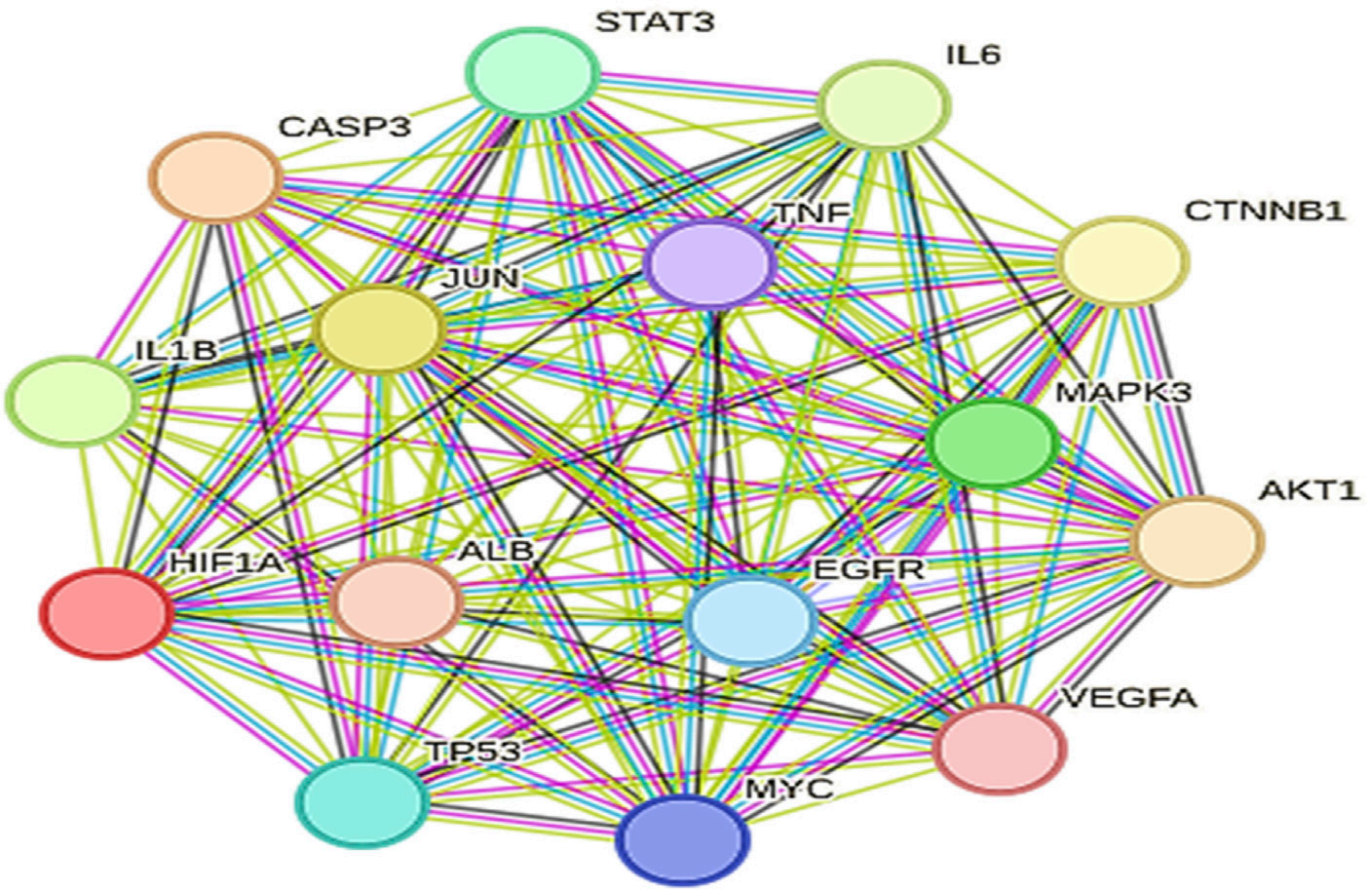
Top 15 significant genes which has been performed by using Cytohubba.

The selection of core targets was based on the network’s topological features, including degree centrality (DC), which measures the number of connections associated with a node, closeness centrality (CC), which calculates the sum of distances from one node to all other nodes, and betweenness centrality (BC), which assesses the importance of a node in terms of shortest paths within the network. These evaluations were performed using the Cyto-NCA plugin. To construct the essential sub-network, the Degree, Closeness, and Betweenness measures of the created networks were further filtered using the CYTOHUBBA plugin and the maximum clique centrality (MCC) scoring technique. In the network target workspace of CYTOHUBBA, computations based on the degree were performed to identify the top 15 significant genes in the network, considering genes with a p-value below a certain threshold. Furthermore, the proteins TP53, STAT3, AKT1, and IL6 were prioritized for molecular docking studies due to their high centrality scores. These high scores across betweenness, closeness, and degree measures indicate their importance within the protein-protein interaction network and suggest their potential as key therapeutic targets (Tables 2, 3; Figure 7).

**TABLE 2:** Top 15 significant genes based on their betweenness, closeness and degrees.

Gene name	Betweeness	Closeness	Degree
TP53	1807.0201493682775	0.9264069264069265	197.0
STAT3	957.8503527532259	0.9184549356223176	195.0
AKTl	905.3734446392816	0.9145299145299145	194.0
IL6	712.842607990732	0.8879668049792531	187.0
VEGFA	697.2263526976875	0.8770491803278688	184.0
TNF	676.0164936870538	0.8734693877551021	183.0
ALB	696.3884688783338	0.8699186991869918	182.0
MYC	826.0072804023529	0.8663967611336032	181.0
JUN	670.0529092860817	0.852589641434263	177.0
EGFR	662.9138584704793	0.8458498023715415	175.0
CTNNBl	683.645313099326	0.84251968503937	174.0
CASP3	529.8929020329398	0.8294573643410853	170.0
HIFlA	509.47708195337566	0.8262548262548263	169.0
ILlB	519.4686905135328	0.8199233716475096	167.0
MAPK3	451.58200981266066	0.8045112781954887	162.0

**TABLE 3:** List of the genes and the proteins encoded.

Sl. NO	Genes	PDB ID of proteins encoded
1	TP53	1TUP
2	STAT3	6TLC
3	AKT1	6S9X
4	IL6	1IL6

### 3.3 Molecular docking simulation

**FIGURE 8:**
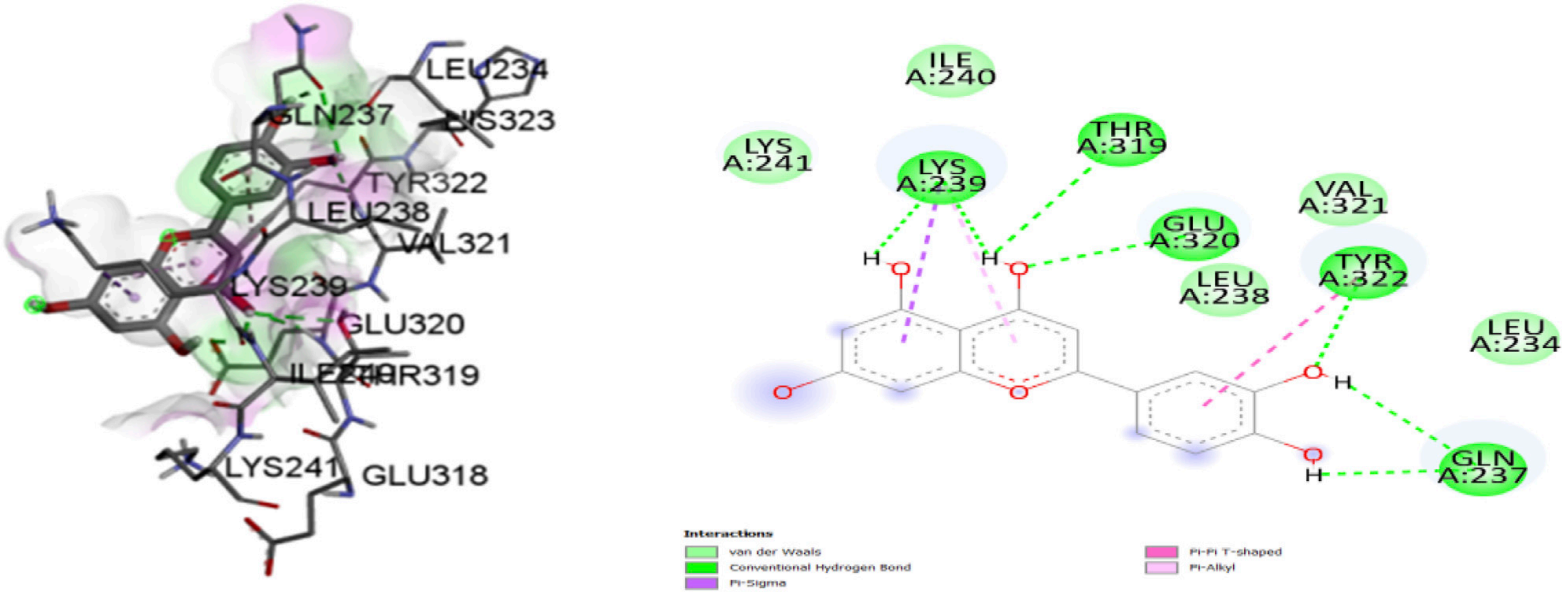
Molecular docking interactions of ligand Luteoline (conformation = −7.8) with 1IL6 protein in 2D and 3D representation.

**FIGURE 9:**
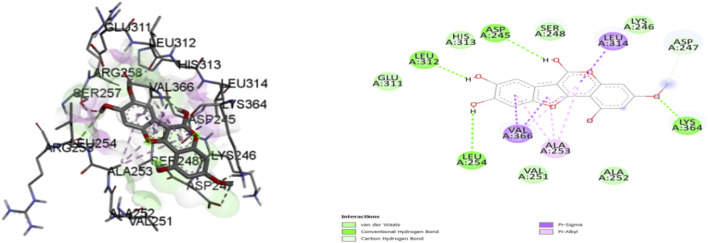
Molecular docking interactions of ligand wedelactone (conformation = −6.7) with 1IL6 protein in 2D and 3Drepresentation.

Based on Molecular docking studies, a list of ligand binding conformations was obtained. The compound’s binding conformation to the selected proteins was found, and the conformation with the lowest binding energy was determined (Table 4). The top 2 compounds Luteolin and Wedelactone with the lowest binding energy and maximum binding affinity, are displayed in the Figures 8, 9, and their respective bonded and non-bonded interaction data are presented in Table 5. Lower binding energy scores indicate stronger protein-ligand binding affinity when compared to higher binding energy values (Foudah et al., 2023). Comparing all three scores and aspects, which includes binding affinity, the number of hydrogen bonds present, and non-bonded interactions, Luteolin ranks first with the lowest scores, followed by Wedelactone.

**TABLE 4:** Table of binding affinity, non-binding interactions, and number of hydrogen bonds of the compounds with the target protein.

Sl. No	Compound name	Protein name	Binding affinity (kcal/mol)	Number of hydrogen bonds	Number of pocket atoms
1	4beta-Hydroxyverazine	1IL6	−7.9	1	12
2	25beta-Hydroxyverazine		−8.0	2	11
3	Linoleic acid		−4.1	1	9
4	Palmitic acid		−4.0	2	8
5	Ricinoleic acid		−4.4	1	5
6	Nicotine		−4.1	1	3
7	Wedellactone		−6.7	4	7
8	beta-Sitosterol		−7.1	2	7
12	2-Formyl-terthienyl		−5.0	1	4
13	alpha-Terthienyl methanol		−5.4	2	7
14	Oleic acid		−3.8	3	8
15	Demethyl wedelolactone		−6.8	2	6
16	Ecliptalbine		−8.0	2	9
17	Pratensein		−8.3	2	7
18	Ursolic acid		−7.8	2	10
19	Pratensein		−7.1	4	2
20	Diosmetin		−7.1	4	2
21	4-Hydroxybenzoic acid		−5.1	2	7
22	Luteolin		−7.8	7	5

**TABLE 5:** Molecular docking interaction details of Luteoline and Wedelactone ligands against 1IL6 protein.

Sl. No	Molecular/compound name	Bonded interactions	Non- bonded interactions
1	Luteolin	LYS:239, THR:319, GLU:320, TYR:322, GLN:237	LYS:241, ILE:240, VAL:321, LEU:238, LEU:234
2	Wedelactone	LEU:312, ASP:245, LYS:364, LEU:254	GLU:311, HIS:313, SER:248, LYS:246, ASP:247, ALA:252, VAL:251

#### 3.3.1 Hydrogen bond interactions

A comprehensive analysis of the binding affinity of Luteoline and Wedelactone to proteins was conducted. According to the research on the luteoline-binding mechanism to 1IL6’s catalytic region, residues LYS-239, THR-319, GLU-320, TYR-322, and GLN-237 are in hydrogen bond contact with the ligand and contribute seven hydrogen bonds. In terms of the Wedelactone-1IL6 binding, LEU-312, ASP-245, LYS-364, and LEU-254 were observed to be involved in hydrogen bond interactions, providing four hydrogen bonds (Shivalingaiah et al., 2022b). The interactions between Luteoline and Wedelactone and 1IL6 demonstrate that both polar and aromatic amino acids are essential for ligand-binding.

#### 3.3.2 Hydrophobic interactions

The influence of hydrophobic interactions on ligand-enzyme interactions is significant. To study the residues of 1IL6 that are engaged in hydrophobic interactions with the ligand, the Discovery Studio visualization program was used. In the Luteoline-1IL6 complex analysis, five hydrophobic bonds were formed with the ligand by LYS-241, ILE-240, VAL-321, LEU-238, and LEU-234. In the Wedelactone-1IL6 complex study, seven hydrophobic bonds were created between the ligand and GLU-311, HIS-313, SER-248, LYS-246, ASP-247, ALA-252, and VAL-251. It is observed that 1IL6 has more residues associated with it compared to other ligands.

The strong hydrogen bonding and hydrophobic interactions of Luteolin suggest a potentially higher inhibitory effect on IL6, which could contribute to anti-inflammatory properties by modulating IL6-mediated signaling pathways. Given IL6’s role in chronic inflammation, autoimmune diseases, and cancer progression, Luteolin’s strong interaction profile may indicate therapeutic potential in these conditions. While Wedelactone exhibits substantial hydrophobic interactions, its lower number of hydrogen bonds suggests a relatively stable but less potent binding with IL6. Nevertheless, its binding affinity still suggests a possible role in IL6 inhibition, albeit to a lesser extent compared to Luteolin. The observed interactions indicate that Luteolin may serve as a stronger lead compound for targeting IL6, with promising implications in diseases where IL6 overexpression plays a role.

### 3.4 Pharmacokinetic properties

In terms of absorption, both compounds showed moderate water solubility, with Luteolin (−3.094) being slightly more soluble than Wedellactone (−3.277). Luteolin demonstrated better intestinal permeability (Caco-2 permeability of 0.096 compared to −0.23 for Wedelolactone). However, Wedelolactone had higher human intestinal absorption (93.753%) than Luteolin (81.13%). Both compounds exhibited low skin permeability (−2.735) and were identified as substrates of P-glycoprotein, which may influence their bioavailability. Neither compound inhibited P-glycoprotein I or II, suggesting minimal risk of efflux-related drug interactions.

For distribution, Luteolin had a higher volume of distribution (VDss of 1.153) than Wedellactone (0.129), indicating broader tissue distribution. The fraction of unbound drug in plasma was higher for Luteolin (0.168) than Wedellactone (0.03), suggesting greater availability of the free drug. Both compounds showed poor BBB permeability, with Wedellactone (−1.353) being slightly less permeable than Luteolin (−0.907). Similarly, CNS permeability was low for both, with values of −2.251 for Luteolin and −2.322 for Wedellactone.

In the metabolism analysis, neither compound was a substrate for CYP2D6. However, Wedellactone was a substrate for CYP3A4, while Luteolin was not. Both compounds inhibited CYP1A2 and CYP2C9 enzymes, indicating potential drug-drug interactions involving these pathways. Neither compound inhibited CYP3A4 or CYP2D6, reducing the likelihood of interactions with these isoforms. The excretion properties showed that Wedellactone had higher predicted total clearance (0.641) compared to Luteolin (0.495), indicating faster elimination. Neither compound was identified as a renal organic cation transporter (OCT2) substrate, suggesting minimal renal transporter-mediated clearance. In terms of toxicity, Luteolin was predicted to be non-mutagenic (negative for AMES toxicity), while Wedellactone was mutagenic (positive for AMES toxicity). Both compounds had comparable maximum tolerated doses (0.499 for Luteolin and 0.546 for Wedelolactone) and were non-hepatotoxic and non-sensitizing to the skin. Luteolin exhibited higher minnow toxicity (3.169) compared to Wedellactone (0.655), indicating potential environmental concerns.

Luteolin is the more promising candidate for drug development due to its balanced absorption, favorable distribution, safer metabolic profile, and lower toxicity. Wedellactone’s mutagenicity and reliance on CYP3A4 metabolism make it less favorable despite its excellent intestinal absorption and clearance (Table 6).

**TABLE 6:** Assessing absorption, distribution, metabolism, excretion, and toxicity profiles of Luteolin and Wedelolactone’s drug-likeness and safety properties.

Property	Model name	Predicted value for luteolin	Predicted value for Wedellactone
Absorption	Water solubility	**−3.094**	**−3.277**
	Caco2 permeability	**0.096**	**−0.23**
	Intestinal absorption (human)	**81.13**	**93.753**
	Skin Permeability	**−2.735**	**−2.735**
	P-glycoprotein substrate	Yes	Yes
	P-glycoprotein I inhibitor	No	No
	P-glycoprotein II inhibitor	No	No
Distribution	VDss (human)	**1.153**	**0.129**
	Fraction unbound (human)	**0.168**	**0.03**
	BBB permeability	**−0.907**	**−1.353**
	CNS permeability	**−2.251**	**−2.322**
Metabolism	CYP2D6 substrate	No	No
	CYP3A4 substrate	No	Yes
	CYP1A2 inhibitior	Yes	Yes
	CYP2C19 inhibitior	No	No
	CYP2C9 inhibitior	Yes	Yes
	CYP2D6 inhibitior	No	No
	CYP3A4 inhibitior	No	No
Excretion	Total Clearance	**0.495**	**0.641**
	Renal OCT2 substrate	No	No
Toxicity	AMES toxicity	No	Yes
	Max. tolerated dose (human)	**0.499**	**0.546**
	hERG I inhibitor	No	No
	hERG II inhibitor	No	No
	Oral Rat Acute Toxicity (LD50)	**2.455**	**2.415**
	Oral Rat Chronic Toxicity (LOAEL)	**2.409**	**2.263**
	Hepatotoxicity	No	No
	Skin Sensitisation	No	No
	*T.Pyriformis* toxicity	**0.326**	**0.303**
	Minnow toxicity	**3.169**	**0.655**

### 3.5 Molecular dynamics simulation studies

The RMSD of the protein-ligand complex illustrates its stability throughout the simulation by identifying whether the ligand remains in the binding pocket. When calculating root mean square distances, the Rg (radius of gyration) considers the rotational axis along with the various masses. It considers the conformation, shape, and folding at each time step throughout the entire trajectory. MSF (mean square fluctuation) focuses on the protein structural areas that deviate the most or least from the mean. SASA (solvent-accessible surface area) assesses the hydrophobic core created when protein-ligand complexes interact. The stability of the ligand during the simulation procedure is also indicated by the ligand RMSD. Moreover, H-bonds are visible during the entire simulation period of the MD study under examination. All intermolecular H-bonds between the ligands and the protein were considered and appropriately displayed during the analysis.

**FIGURE 10:**
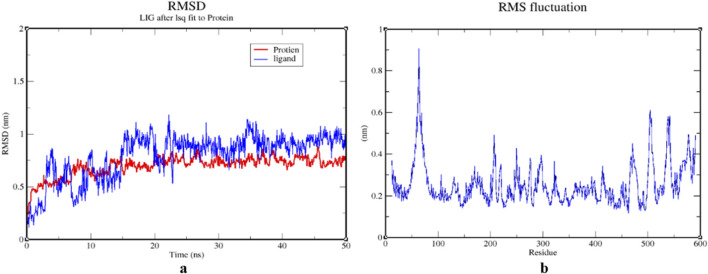
**(A)** RMSD graphs of 1IL6 and its complex with Luteoline for time trajectory from 0 ns to 50 ns. **(B)** RMSF. **(A)** RMSD graphs of 1IL6 and its complex with Luteolin over a 50 ns simulation trajectory, indicating the stability of the protein-ligand interaction. A steady RMSD value suggests a stable binding interaction, whereas large fluctuations indicate conformational changes. **(B)** RMSF plot, highlighting structural flexibility and dynamic regions of the protein. Higher RMSF values correspond to more flexible regions, while lower values indicate structurally stable regions, crucial for ligand binding and overall protein stability.

The stability of the 1IL6 and Luteolin was assessed through 50 ns MDS. These simulations provided detailed information on the binding interaction of the docking complex with a system containing water molecules, as well as information on temperature and pressure. In all appropriate binding postures, the complex exhibited an acceptable RMSD value of 3. According to the RMSD plots, the protein and ligand appeared to be more stable in the 50 ns simulation study. During the simulation procedure, the RMSD value for the ligand appears to proportionally increase in the first 25 ns and then mostly fluctuates between 0.7 and 1.3 nm. After 25 ns and up to 50 ns, a slight fluctuation is observed in the ligand. The protein RMSD trajectory initially fluctuated between 0–10 ns, with a slight fluctuation between 10–20 ns. After 20 ns, the protein RMSD trajectory slowly rises until the end of the simulation. There were no unusual swings in the RMSD for the protein during the simulation (Figure 10A).

**FIGURE 11:**
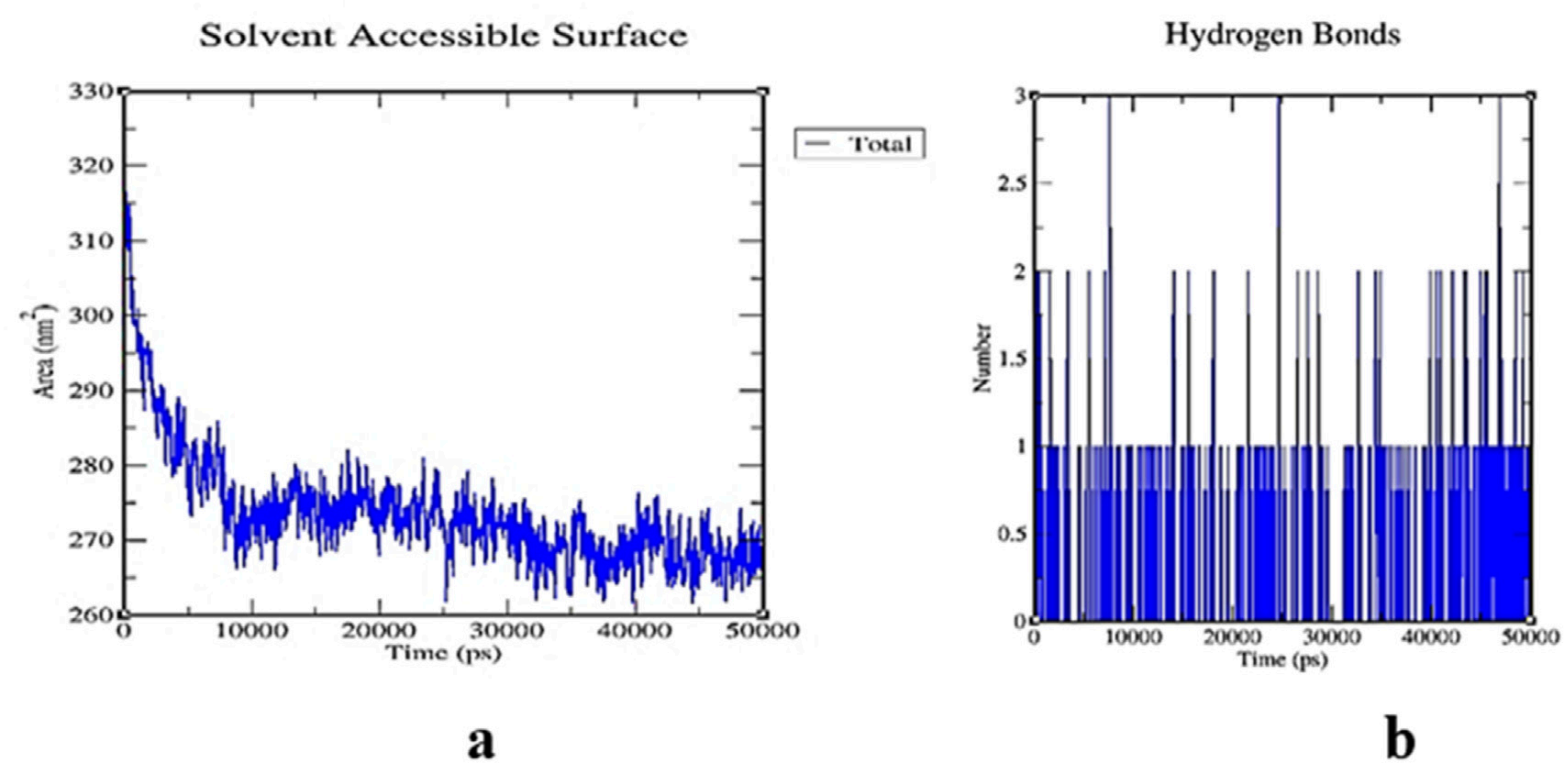
**(A)** Hydrogen bond plot depicting the number of hydrogen bonds formed between 1IL6 and Luteolin throughout the 50 ns simulation, demonstrating the persistence and strength of ligand binding. A stable hydrogen bond network suggests a strong interaction with the target protein. **(B)** SASA plot of the protein-ligand complex, illustrating changes in the hydrophobic core exposure upon ligand binding. A consistent SASA range (90–95.5 nm^2^) indicates regular complex formation and minimal structural disruption, reinforcing the potential of Luteolin as a stable IL6 inhibitor. The estimated range for protein and protein-ligand complex SASA plots was 90–95.5 nm2, demonstrating regularity in complex formation. Further demonstrating the long-term viability of Luteoline was the ligand RMSD, which leveled after 20 nanoseconds and approached equilibrium at 0.5 nm. In Addition, the maximum number of ligand hydrogen bonds of seven had been expected.

The counterplots of RMSF indicate significant variations throughout the simulation, in contrast to RMSD trajectories. A description of macromolecule stead y-state and heterogeneity is provided by RMSF. The ubiquitous intermolecular hydrogen bonds are essential for protein folding and interactions with ligands. It was determined throughout the 50 ns simulation period how stable the hydrogen bond network created in the protein-ligand complex was. complexes’ overall hydrogen bond count over time at 500 K. The 1IL6-Luteoline complex displayed the necessary number of hydrogen bonds throughout the simulation, demonstrating its strong and stable hydrogen bonding (Figures 10B, 11A, B).

### 3.6 Binding free energy calculations

Various energy metrics such as Van der Waal’s, electrostatic, polar solvation, SASA, and binding energies are utilized to measure the extent of ligand-target protein binding interactions during molecular dynamics simulations. In this study, the electrostatic energy was primarily used to construct the protein-ligand combination. Van der Waal’s energy, SASA energy, and binding energy came next. Polar solvation energy was predicted with no contribution to the protein-ligand complex formation, as the values appeared positive. The 1IL6 complexed with Luteolin was predicted with the highest binding affinity and hence was considered for binding energy calculation studies. In addition, the protein-ligand complex standard deviations were calculated. A lower standard deviation means the data values are closer to the mean (or expected value), whereas a high standard deviation means the data values are spread out over a wider range. However, in the complex, there were no high standard deviations. This indicates that Luteolin binds to the protein with high binding affinity and stable interaction. The binding free energy calculations of the protein-ligand complex have been given in Table 7.

**TABLE 7:** Binding free energy calculations of 1IL6 target protein complexed with Luteolin.

Categories	1IL6 - Luteolin complex	
	Values (kj/mol)	Standard deviation (kj/mol)
Van der Waal’s energy	−179.746	+/− 131.137
Electrostatic energy	−25.672	+/− 39.198
Polar solvation energy	57.164	+/− 43.852
SASA energy	−11.120	+/− 10.227
Binding energy	−197.440	+/− 145.163

## 4 Discussion

In the current study, a comprehensive approach was taken to investigate the network of PPI and C-T relationships. The list of common genes identified for AD and GBM was input into the STRING database to establish an interaction network. The network was constructed using a confidence level of 0.09 and filtered for *H. sapiens*, specifically excluding dissociation targets from the list of target genes. The resultant PPI network was analyzed using the STRING database, which offers a reliable way to visualize molecular networks and understand the relationships between genes involved in disease mechanisms. A total of 13 clusters were obtained using the CLUSTER-ONE algorithm from the Cytocluster plugin, which employs overlapping neighborhoods for clustering weighted graphs. Among these clusters, only 8 clusters with p-values less than 0.05 were considered for further analysis, indicating significant interactions that may play a crucial role in the pathophysiology of both AD and brain cancer (Tohma et al., 2019).

Gene prediction involved the selection of core targets based on topological features within the constructed PPI network. The key features considered for predicting core genes were DC, CC, and BC. The degree, closeness, and betweenness measures were subsequently used to filter the top 15 significant genes from the network using the CYTOHUBBA plugin. For instance, TP53, a well-known tumor suppressor, exhibits the highest betweenness centrality, indicating its critical role in mediating interactions between other genes in the network. Similarly, STAT3 and AKT1, both involved in cell signaling and survival pathways, show high centrality scores across all three measures. This suggests that these genes not only interact with numerous other genes but also play a crucial role in information flow and overall network influence. Other notable genes in the top 15 include IL6, a pro-inflammatory cytokine; VEGFA, a key regulator of angiogenesis; and TNF, another pro-inflammatory cytokine, all of which have established roles in the pathogenesis of both AD and GBM. The top four genes were further analyzed for their associated proteins and interactions, and their complex analysis provided valuable insights into the molecular pathways that might be targeted in the treatment of both AD and GBM.

Molecular docking simulations were employed to assess the binding affinity of selected ligands with proteins involved in AD and GBM. Among the tested compounds, Luteolin and Wedelactone emerged as the top two ligands, exhibiting the lowest binding energies and maximum binding affinities. The hydrogen bond interactions between the ligands Luteolin and Wedelactone and their target protein, 1IL6, were critically analyzed to understand the nature of these interactions. Luteolin formed seven hydrogen bonds with key residues of 1IL6. These interactions are vital for the ligand-protein stability, suggesting a high degree of specificity in binding. On the other hand, Wedelactone formed four hydrogen bonds with residues which, though fewer in number, still contribute significantly to the protein-ligand binding. The analysis revealed that both ligands formed strong interactions with polar and aromatic amino acids, highlighting the importance of these residues in stabilizing the ligand binding, which could be pivotal for therapeutic efficacy. Hydrophobic interactions are essential in stabilizing ligand-protein complexes. The study focused on the hydrophobic interactions between the ligands and the protein 1IL6. Luteolin formed five hydrophobic interactions with key protein residues. These interactions are crucial for the overall binding stability, providing additional non-polar interaction forces that contribute to the stability of the protein-ligand complex. Similarly, Wedelactone exhibited seven hydrophobic interactions with residues. The comparison of the two ligands indicated that Luteolin exhibited slightly fewer hydrophobic interactions but still maintained a strong binding profile (Vinusha et al., 2022). The importance of hydrophobic interactions in drug binding underscores their role in drug efficacy and stability.

Both compounds showed moderate water solubility, with Luteolin slightly outperforming Wedelactone in this regard. Luteolin also exhibited better intestinal permeability, suggesting that it may be absorbed more efficiently in the human gastrointestinal tract. Based on all the pharmacokinetic properties, Luteolin appeared to be the more promising candidate for drug development, offering a safer metabolic profile and more favorable drug-likeness. Despite their strong molecular docking interactions with IL6, a key mediator of neuroinflammation, Luteolin and Wedelolactone’s low permeability suggests that systemic administration may not achieve therapeutically relevant concentrations in the CNS. This limitation presents a challenge for their direct use in brain-related disorders, necessitating innovative strategies to enhance their bioavailability and efficacy.

To overcome these challenges, nanotechnology-based drug delivery systems offer a promising solution. Encapsulation of Luteolin and Wedelolactone in nanoparticles (NPs) can significantly improve their stability, solubility, and targeted delivery to the brain, allowing them to cross the BBB more effectively. Nanoparticles provide multiple advantages in drug delivery, particularly for brain-targeted therapy. Enhanced BBB penetration ensures efficient drug transport. Controlled and sustained drug release extends the half-life of encapsulated compounds, allowing for prolonged therapeutic effects. Improved bioavailability protects Luteolin and Wedelolactone from metabolic degradation, increasing drug stability and solubility. Targeted drug delivery using ligand-functionalized nanoparticles ensures that drugs are directed precisely to affected brain regions. Reduced systemic toxicity minimizes off-target effects, decreasing toxicity risks associated with high systemic drug concentrations. Several nanoparticle-based approaches can be employed to enhance CNS drug delivery. Lipid-based nanoparticles (LNPs) and polymeric nanoparticles (PNPs) improve the solubility and metabolic stability of phytocompounds. Functionalizing nanoparticles with low-density lipoprotein (LDL) receptors or transferrin receptors can facilitate receptor-mediated transcytosis across the BBB. Solid lipid nanoparticles (SLNs) serve as carriers that protect Luteolin and Wedelolactone from metabolic degradation and allow sustained release, increasing their availability in the brain. Liposomes and nanoemulsions encapsulate phytochemicals and facilitate passive diffusion across the BBB, improving their therapeutic concentration in the brain. Modifying Luteolin’s hydroxyl (-OH) groups by methylation or acetylation can reduce hydrogen bonding and increase lipophilicity, facilitating passive diffusion across the BBB. Additionally, prodrug strategies, where Luteolin is esterified or conjugated with lipid carriers, could enhance CNS penetration and undergo enzymatic conversion into the active form upon reaching the brain. Another promising strategy involves glycosylation and nanoformulation, which have been shown to enhance flavonoid stability, solubility, and targeted delivery. Luteolin derivatives, such as Luteolin-7-O-glucoside, have demonstrated improved bioavailability while retaining neuroprotective and anti-tumor effects.MD simulations were employed to assess the stability and behavior of the 1IL6-Luteolin complex over time.,with the ligand showing minor fluctuations after 25 ns, indicating that it had reached an equilibrium conformation. and thus confirmed that the 1IL6-Luteolin complex is stable, with minimal structural deviations, reinforcing Luteolin’s potential as a strong ligand for therapeutic applications.

Binding free energy calculations were performed to evaluate the strength of the interaction between Luteolin and the target protein 1IL6. The negative values of Van der Waals and electrostatic energies indicate favorable binding interactions, while the positive polar solvation energy suggests that solvation effects may not significantly contribute to the binding. The overall binding energy calculation, along with stable interactions, further supports the suitability of Luteolin as a potential therapeutic agent for AD and GBM.

While Luteolin exhibits anti-inflammatory, antioxidant, and neuroprotective properties, its therapeutic potential can be further enhanced by combination therapies with other compounds. A multi-target approach can improve treatment outcomes for complex neurodegenerative and oncological disorders. Luteolin and curcumin can be combined to synergistically inhibit IL6-related neuroinflammation, enhancing therapeutic efficacy in AD and GBM. Luteolin and resveratrol can work together to reduce oxidative stress and neuronal damage. Luteolin and quercetin may provide a more comprehensive neuroprotective strategy by targeting amyloid-beta aggregation. Luteolin and EGCG (Epigallocatechin Gallate), found in green tea, could enhance Luteolin’s anti-neuroinflammatory and anti-cancer effects. In brain cancer treatment, Luteolin and doxorubicin could be combined to enhance cytotoxic effects while reducing the toxicity associated with high chemotherapy doses. Combination therapies involving Luteolin with other neuroprotective or anticancer compounds may enhance therapeutic efficacy through synergistic effects on multiple pathological pathways. By integrating nanotechnology and combination strategies, Luteolin-based therapies could be more effectively translated into clinical applications for treating neurodegenerative diseases and brain cancer.

Kollur et al. (2021) explained luteolin’s established cytotoxic mechanisms in breast cancer, involving ROS generation, DNA damage signaling, NF-κB inhibition, p38 activation, and apoptosis induction, offer a starting point for exploring its potential in AD and GBM, although the specific pathophysiology of each disease suggests distinct avenues for its action. In AD, luteolin’s ROS generation, while potentially cytotoxic, might also stimulate autophagy, aiding in Aβ aggregate removal. Its NF-κB inhibition could dampen neuroinflammation, a key driver of AD progression. Furthermore, luteolin’s impact on mitochondrial membrane potential and cytochrome C release could induce apoptosis in dysfunctional neurons, while its potential antioxidant properties might offer neuroprotection. In GBM, luteolin’s cytotoxic effects, particularly apoptosis induction through multiple pathways, are directly relevant to treatment. NF-κB inhibition could suppress glioblastoma growth, while p38 activation might sensitize cells to chemotherapy. It's also crucial to investigate potential anti-angiogenic properties of luteolin, given the importance of angiogenesis in glioblastoma. A comprehensive evaluation of Luteolin’s potential off-target effects is crucial for understanding its safety and therapeutic potential, especially as a dual-target treatment for AD and GBM. While Luteolin demonstrates promising binding affinity to IL6, it may also interact with other proteins, leading to unintended effects. To assess this, a detailed analysis of Luteolin’s binding profiles with additional proteins could be conducted using molecular docking, network pharmacology, and protein-ligand interaction prediction tools. Screening against a broader protein database can reveal possible off-target interactions with enzymes, receptors, or signaling molecules that might cause adverse effects such as toxicity, immune modulation, or altered pharmacokinetics.

These are speculative hypotheses, and further research tailored to AD and glioblastoma is essential to validate them. Studies should examine luteolin’s effects on relevant models, investigating endpoints like Aβ aggregation, tau phosphorylation, neuroinflammation, tumor growth, and angiogenesis to understand its precise interactions with disease-specific molecular pathways and determine its therapeutic potential. Luteolin has shown low toxicity in short-term studies, but its long-term effects remain unclear, especially when used as a treatment for chronic, progressive diseases. Evaluating potential cumulative toxicity, including organ-specific toxicity, and assessing its interaction with other drugs commonly used in AD or GBM (such as anti-inflammatory agents, antipsychotics, or chemotherapy drugs) is critical. *In vivo* studies focused on chronic administration could shed light on any long-term risks, such as cumulative oxidative stress or immune system suppression. Moreover, tolerability studies should explore whether Luteolin’s bioavailability and efficacy change over prolonged use and whether its therapeutic windows remain safe.

## 5 Conclusion and future prospects

The present study offers a robust multi-faceted approach to exploring the therapeutic potential of Luteolin for AD and GBM, demonstrating its strong promise as a dual-target therapeutic agent. Through the integration of network pharmacology, molecular docking, pharmacokinetic analysis, and molecular dynamics simulations, we have gathered compelling evidence supporting Luteolin’s efficacy in modulating key molecular pathways involved in both diseases. The identification of 13 clusters in the PPI network and the subsequent focus on the top 15 central genes highlight the interconnected molecular mechanisms driving the pathophysiology of AD and brain cancer.

Luteolin, identified as the top ligand through molecular docking simulations, exhibited strong binding affinity with target proteins, particularly 1IL6, forming stable interactions through hydrogen bonds and hydrophobic forces. These interactions, combined with favorable pharmacokinetic properties, such as better intestinal permeability and a favorable metabolic profile, position Luteolin as a promising candidate for further drug development. The results from molecular dynamics simulations further confirmed the stability of the Luteolin-protein complex, reinforcing its potential therapeutic applications. The binding free energy calculations, indicating a high binding affinity, further support Luteolin’s viability as a candidate for targeting both Alzheimer’s and brain cancer. Despite challenges such as limited BBB permeability, Luteolin’s multifaceted mechanism of action, favorable toxicity profile, and promising pharmacokinetic characteristics make it an attractive lead compound for the development of novel therapeutic strategies in treating neurodegenerative diseases and brain cancers. Future clinical studies and optimization of Luteolin’s bioavailability could pave the way for its translation into a viable treatment for these challenging conditions.

The promising findings from this study pave the way for several future research directions and clinical applications. First, further refinement of Luteolin’s pharmacokinetic profile is essential to enhance its bioavailability, particularly in overcoming the challenges associated with BBB penetration. Additionally, while this study identified Luteolin as a promising therapeutic candidate, further preclinical and clinical investigations are needed to evaluate its efficacy and safety *in vivo*. Animal model studies will be crucial to assess the real-world applicability of Luteolin, particularly in terms of long-term treatment outcomes and potential side effects, the C57BL/6 mouse model provides a versatile and well-established system for evaluating Luteolin’s therapeutic potential in both neurodegenerative and oncological application.Comprehensive clinical trials are also necessary to confirm its therapeutic benefits in human populations, focusing on optimal dosages, administration routes, and potential combination therapies with other anticancer or neuroprotective agents. The use of network pharmacology and systems biology in this study has underscored the complex molecular networks involved in both Alzheimer’s and brain cancer, opening up avenues for exploring additional polypharmacological approaches. Future research could aim to identify additional bioactive compounds that may synergize with Luteolin, providing a more comprehensive treatment strategy. Combining multiple therapeutic agents targeting different aspects of disease pathophysiology could enhance treatment efficacy and reduce the potential for resistance or relapse.

## Data Availability

The original contributions presented in the study are included in the article/supplementary material, further inquiries can be directed to the corresponding authors.

## Abbreviations

AD, Alzheimer’s Disease; GBM, Glioblastoma; TP53, Tumor protein p53; STAT3, Signal Transducer and Activator of Transcription 3; AKT1, AKT serine/threonine kinase 1; IL6, Interleukin-6; ADMET, Absorption, Distribution, Metabolism, Excretion, and Toxicity; PPI, Protein-Protein Interaction; CNS, Central Nervous System; BBB, Blood-Brain Barrier; MD, Molecular Docking; MDS, Molecular Dynamic Simulations; RMSD, Root Mean Square Deviation; RMSF, Root Mean Square Fluctuation; SASA, solvent-accessible surface area; PDB, Protein Data Bank; CASTp, Computed Atlas of Surface Topography of Proteins; MM-PBSA, Molecular Mechanics/Poisson-Boltzmann Surface Area; DC, Degree centrality; CC, Closeness Centrality; BC, Betweenness Centrality; NPs, Nanoparticles; LNPs, Lipid-based nanoparticles; PNPs, Polymeric nanoparticles; LDL, Low-density lipoprotein; SLNs, Solid lipid nanoparticles.
